# A genome-wide screen for resilient responses in growing pigs

**DOI:** 10.1186/s12711-022-00739-1

**Published:** 2022-07-04

**Authors:** Houda Laghouaouta, Lorenzo Fraile, Rafael Suárez-Mesa, Roger Ros-Freixedes, Joan Estany, Ramona Natacha Pena

**Affiliations:** Department of Animal Science, University of Lleida-Agrotecnio-CERCA Center, 25198 Lleida, Catalonia Spain

## Abstract

**Background:**

There is a growing interest to decipher the genetic background of resilience and its possible improvement through selective breeding. The objective of the present study was to provide new insights into the genetic make-up of resilience in growing pigs by identifying genomic regions and candidate genes associated with resilience indicators. Commercial Duroc pigs were challenged with an attenuated Aujeszky vaccine at 12 weeks of age. Two resilience indicators were used: deviation from the expected body weight at 16 weeks of age given the growth curve of non-vaccinated pigs (∆BW) and the increase in acute-phase protein haptoglobin at four days post-vaccination (∆HP). Genome-wide association analyses were carried out on 445 pigs, using genotypes at 41,165 single nucleotide polymorphisms (SNPs) and single-marker and Bayesian multiple-marker regression approaches.

**Results:**

Genomic regions on pig chromosomes 2, 8, 9, 11 (∆BW) and 8, 9, 13 (∆HP) were found to be associated with the resilience indicators and explained high proportions of their genetic variance. The genomic regions that were associated explained 27 and 5% of the genetic variance of ∆BW and ∆HP, respectively. These genomic regions harbour promising candidate genes that are involved in pathways related to immune response, response to stress, or signal transduction (*CD6*, *PTGDR2*, *IKZF1*, *RNASEL* and *MYD88*), and growth (*GRB10* and *LCORL*).

**Conclusions:**

Our study identified novel genomic regions that are associated with two resilience indicators (∆BW and ∆HP) in pigs. These associated genomic regions harbour potential candidate genes involved in immune response and growth pathways, which emphasise the strong relationship between resilience and immune response.

**Supplementary Information:**

The online version contains supplementary material available at 10.1186/s12711-022-00739-1.

## Background

Resilience can be defined as the ability of animals to maintain their production in spite of internal and external stressors that might occur during their productive life [1]. Selective breeding for improved resilience could provide disease-resistant or disease-tolerant animals with more robust phenotypes [2], which would reduce economic losses and increase profitability and sustainability of production systems. However, one of the main limitations is that there is no consensus on how to measure resilience and little is known about its genetic background in different species. Therefore, defining novel resilience indicators and understanding their genetic basis is an essential first step for the improvement of resilience through genetic selection.

In order to be able to measure resilience in livestock species, resilience indicators have been elaborated based on productivity-related traits such as body weight (BW) in chickens [3], litter size in rabbits [4], feed intake in pigs [5], and milk yield in cattle [6]. Resilient animals have steady production levels, with small fluctuations due to environmental challenges. Other indicators based on immune-related traits, such as natural antibody levels in pigs [7], have also been proposed because of their role in the first line of defence against pathogens. In previous work, we proposed two novel resilience indicators in young pigs, i.e. the deviation from the expected body weight (∆BW) and the increase in acute-phase protein haptoglobin (∆HP) after applying a common vaccine challenge [8]. Pigs that maintained their productivity (high ∆BW) and had a low activation of haptoglobin (low ∆HP) were considered resilient, whereas pigs that were highly affected by the perturbations were considered susceptible. We showed that these resilience indicators were genetically controlled, with low to moderate heritabilities but substantial variability in the studied population, which indicated that they could be improved through selective breeding.

Information about the molecular mechanisms that underlie resilience is scarce and depends also very much on the resilience indicator used. A few genome-wide association studies (GWAS) have assessed the molecular genetic basis of resilience. These include analysis of resilience indicators such as antibody levels [7, 9] and health-related traits in pigs [10] and the environmental variance of litter size in rabbits [11]. These studies revealed potential candidate genes that are mainly involved in immune and inflammatory responses, thus corroborating the strong relationship between resilience and the immune system [12]. In addition, a number of studies have focused mainly on disease susceptibility and genetic resistance to specific pathogens. In pigs, two pathogens that have been studied in depth to identify DNA variants that are associated with resilient responses are the porcine reproductive and respiratory syndrome (PRRS) virus [13–15] and *Actinobacillus pleuropneumoniae* [16, 17]. Although these studies have identified DNA variants that contribute to lowering the impact of specific infections, to date no markers have been associated with resilient indicators without the presence of overt diseases under field conditions.

In this study, we used the previously defined resilience indicators ∆BW and ∆HP in order to identify genomic regions that are associated with resilience in growing pigs and elucidate its genetic background.

## Methods

### Animals and phenotypes

The resilience experiment that provided the data for this study has been described in full detail in a previous report [8]. Briefly, at 10 weeks of age (71.4 ± 2.4 days), 540 commercial Duroc barrows were allocated to five fattening batches of 104–111 pigs each. Pigs were progeny of 49 sires and 198 dams, and were reared under the same conditions with ad libitum access to commercial diets. At 12 weeks of age (85.6 ± 2.4 days), 445 of these pigs were intramuscularly challenged with 2 mL (≥ 10^5.5^ TCID_50_) of an attenuated Aujeszky vaccine (Auskipra, Laboratorios Hipra, Amer, Girona) and 95 were inoculated with phosphate-buffered saline (control pigs). Challenged and control pigs were evenly distributed among batches. Pigs were weighed at − 14, 0, and 28 days post-vaccination (dpv) and bled at 4 dpv to analyse their haptoglobin concentration. In addition, 41 challenged and 40 control pigs were bled at 0 dpv to establish the basal level of haptoglobin in each fattening batch.

#### Deviation from the expected growth curve

Body weight data from control pigs were analysed to establish the control growth curve in the absence of the vaccine challenge, as described by Laghouatouta et al. [8]. For each challenged pig, the expected BW at 28 dpv (i.e., 16 weeks of age, approximately) was estimated using the control growth curve in order to calculate the difference between the observed and the expected BW (ΔBW). The average ∆BW of the challenged pigs was − 0.68 (3.64) kg, indicating that the observed BW of challenged pigs was smaller than the expected BW at 16 weeks of age and that there was a wide variability in this trait (see Additional file 1: Figure S1) [8].

#### Increase in haptoglobin after the vaccine challenge

The concentration of the acute-phase protein haptoglobin in serum was quantified at 0 and 4 dpv using a spectrophotometric method, as described by Saco et al. [18]. The increase in haptoglobin at 4 dpv (∆HP) was calculated as the difference between the pig’s haptoglobin concentration at 4 dpv and the basal level of its fattening batch. Average ∆HP of challenged pigs was + 0.03 (0.7) mg/mL (see Additional file 1: Figure S1). The haptoglobin concentration at 4 dpv was higher than the basal level, with a large variation in the vaccinated group [8].

### Genotypes and quality control

Genomic DNA was isolated using the standard phenol/chloroform method [19]. DNA samples were genotyped with the GeneSeek GGP Porcine HD array (Illumina, San Diego, CA, USA), which features ~ 70K single nucleotide polymorphisms (SNPs). Quality control was performed using the PLINK v1.9 software [20]. Individuals with a missing genotype frequency higher than 0.1 and SNPs with a minor allele frequency lower than 0.05, a genotyping rate lower than 0.95, or an unknown position in the pig genome assembly *Sscrofa 11.1* were excluded from the dataset. After quality control, the dataset comprised 41,165 SNPs and 445 individuals.

### Genome-wide association study

Association analyses for the phenotypes ∆BW, ∆HP, and BW at 28 dpv (BW_28_) were carried out using both a single-marker regression (SMR) approach and a Bayesian multiple-marker regression (Bayes B) approach, using the GEMMA [21] and the GenSel [22] softwares, respectively. SMR does not take linkage disequilibrium between SNPs into account and the effects of significant SNPs is overestimated [23]. Bayes B evaluates the association between a given phenotype and a large set of SNPs simultaneously [22]. However, it does not consider the population structure within the pedigree because the GenSel software does not allow implementation of the genomic relationship matrix in the model. Hence, the first four components of a principal component analysis of the genotypes were fitted as covariates to account for the pedigree structure in the Bayes B approach.

### Single-marker regression

Single-marker regression was performed to evaluate the association between the phenotypes and each SNP, using the following univariate linear mixed model:

$${\mathbf{y}}={\mathbf{X}}{\mathbf{b}}+{\mathbf{z}}_{\text{j}}{\upbeta }_{\text{j}}+{\mathbf{W}}{\mathbf{u}}+{\mathbf{e}},$$

where $\mathbf{y}$ is the vector of phenotypic observations (∆BW, ∆HP, or BW_28_); $\mathbf{X}$ is the incidence matrix for systematic effects; $\mathbf{b}$ is the vector of systematic effects, which included the intercept and batch (5 levels); ${\mathbf{z}}_{\text{j}}$ is the vector of genotypes of the $\text{j}$-th SNP coded as 0 and 2 for homozygotes and 1 for heterozygotes (missing genotypes were replaced by the average value of the population); ${\upbeta }_{\text{j}}$ is the allele substitution effect of the $\text{j}$-th SNP; $\mathbf{W}$ is the incidence matrix for polygenic effects; $\mathbf{u}$ is the vector of polygenic effects; and $\mathbf{e}$ is the residual term. Polygenic effects and residuals were assumed to be distributed as $\mathbf{u}\sim N({\mathbf{0}},\mathbf{K}{\upsigma }_{\text{u}}^{2})$ and $\mathbf{e}\sim N({\mathbf{0}},\mathbf{I}{\upsigma }_{\text{e}}^{2})$, where $\mathbf{K}$ is the genomic relationship matrix, ${\upsigma }_{\text{u}}^{2}$ is the additive genetic variance, $\mathbf{I}$ is an identity matrix, and ${\upsigma }_{\text{e}}^{2}$ is the residual variance. Due to the limited sample size and the polygenic nature of the studied trait, SNPs with a suggestive P-value lower than 0.0001 were considered as significantly associated with the trait.

### Bayes B

Bayesian multiple marker regression was carried out using the following Bayes B model to evaluate the association between the phenotype and all SNPs simultaneously:

$${\mathbf{y}}={\mathbf{X}}{\mathbf{b}}+{\sum}_{\text{j}=1}^{\text{k}}{\mathbf{z}}_{\text{j}}{\upalpha}_{\text{j}}{\updelta}_{\text{j}}+{\mathbf{e}},$$

where $\mathbf{y}$ and $\mathbf{X}$ are the same as above; $\mathbf{b}$ is the vector of systematic effects, which included batch (5 levels) and the four first principal components of the principal component analysis of the genotypes, which explained 9.6% of the total variance, as covariates; $\text{k}$ is the number of SNPs that passed the quality control; ${\mathbf{z}}_{\text{j}}$ is the vector of coded genotypes (missing genotypes were replaced by the average value of the population); α_j_ is the allele substitution effect of the $\text{j}$-th SNP; ${\updelta }_{\text{j}}$ is a random binary variable, representing inclusion (${\updelta }_{\text{j}}$ = 1) of the $\text{j}$-th SNP with prior probability $1-\pi$ and its exclusion (${\updelta }_{\text{j}}$ = 0) with prior probability $\pi$ in the model fitted in each iteration of the Monte Carlo Markov chain (MCMC); and $\mathbf{e}$ is the residual term. Due to the limited sample size, parameter $\pi$ was set to 0.998 to increase the detection power of associated SNPs. Thus, each iteration of the MCMC included approximately 82 SNPs. Priors for the systematic effects were flat. Priors for the variance components of the phenotypes were retrieved from previous work [8]. The unknowns in the model were estimated using marginal posterior distributions. An MCMC of 500,000 samples with a burn-in of 100,000 and an output frequency of 40 iterations was used. In total, 2351 non-overlapping 1-Mb windows across the genome were defined, with an average of 17.5 SNPs per window and the genomic variance explained by each window was estimated using the posterior distribution of the genomic variance explained by SNPs within that window.

Bayes factors (BF) were estimated as the ratio between the posterior odds ratio and the prior odds ratio to assess the association between the phenotypes and each SNP, as:

$${\text{BF}}_{\text{j}}=\frac{posterior\,odds\,ratio}{prior\,odds\,ratio}=\frac{{\widehat{\text{p}}}_{\text{j}}/(1-{\widehat{\text{p}}}_{\text{j}})}{(1-\pi )/\pi },$$

where ${\widehat{\text{p}}}_{\text{j}}$ is the posterior probability of the $\text{j}$-th SNP being included in the model at a given iteration of the MCMC and $\pi$ is the prior probability of that SNP having zero effect ($\pi$ = 0.998). As suggested by Kass and Raftery [24], associations were considered strong if the BF was greater than 10.

### Associated regions and candidate genes

Manhattan plots of the GWAS results for ∆BW, ∆HP and BW_28_ were generated using the *ggplot2* package [25]. To avoid false-positive SNPs, only SNPs that were detected with both methods were identified as associated with the phenotype. One-Mb genomic regions on either side of each associated SNP were considered as associated. All genes within the associated genomic regions were retrieved from the Ensembl database [26] using the *Sscrofa11.1* build as the reference genome. Gene functions were further investigated using the DAVID database [27] and a literature search.

## Results

### Body weight deviation from expected growth after the vaccine challenge

**Fig. 1:**
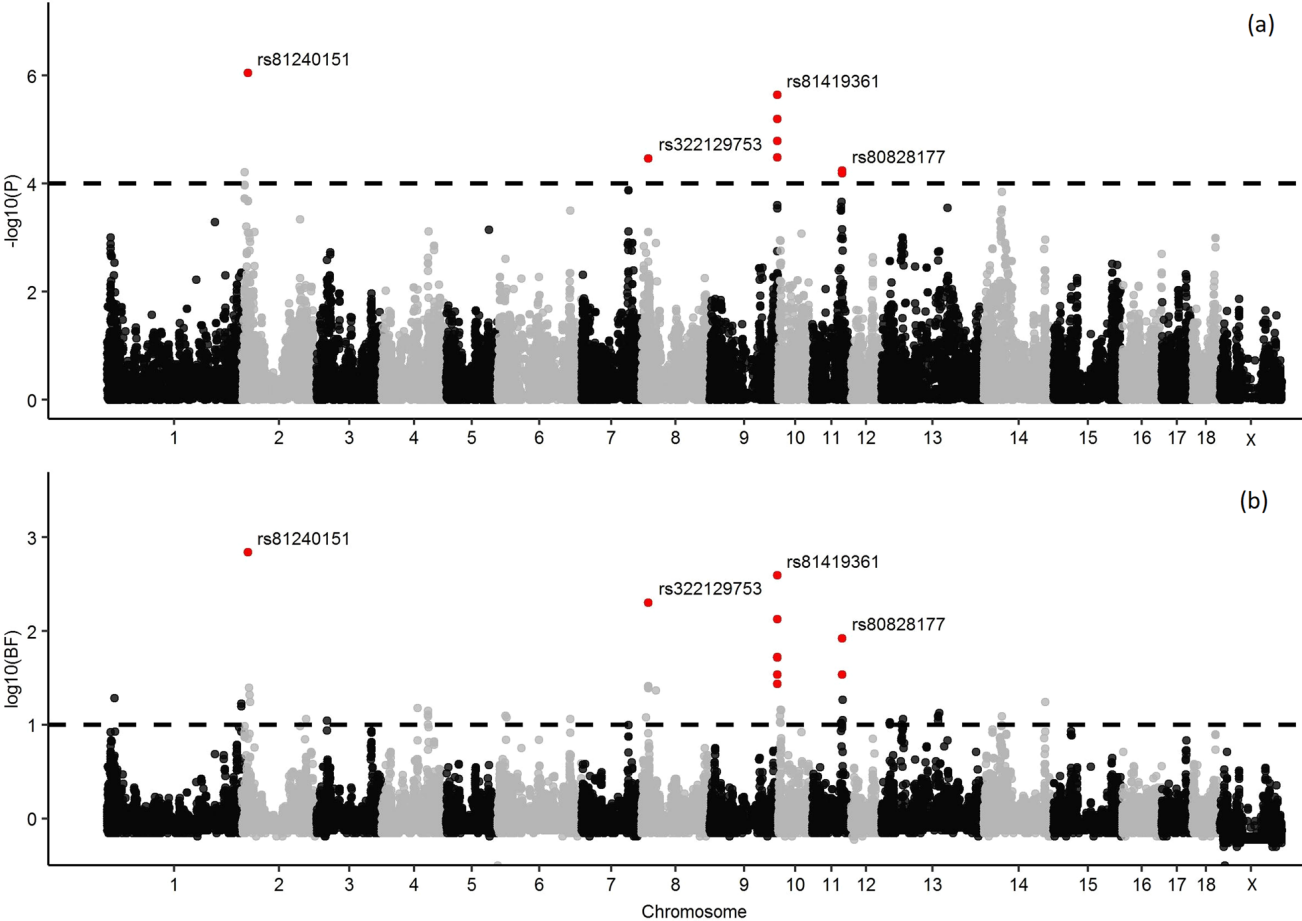
Manhattan plots for the genome-wide association analysis of the deviation of body weight from the expected growth at 16 weeks of age, following vaccination based on **a** single marker regression and **b** Bayesian multiple marker regression. The black dashed line represents the threshold of 0.0001 for p-values (**a**) and of 10 for the Bayes factor (**b**). Associated SNPs are highlighted in red

For ∆BW, SMR identified 11 associated SNPs, on *Sus scrofa* chromosomes (SSC) SSC2, 8, 9, and 11 (Fig. 1a) and (see Additional file 2: Table S1), while Bayes B identified 62 associated SNPs, on SSC1, 2, 3, 4, 6, 7, 8, 9, 10, 11, 13, and 14 (Fig. 1b) and (see Additional file 2: Table S2). Ten of the detected SNPs were in common between the two methods and were deemed to be associated (Table 1). These ten SNPs were located in four genomic regions, on SSC2 (10.7–12.7 Mb), SSC8 (12.8–14.8 Mb), SSC9 (135.8–138.7 Mb), and SSC11 (59.3–61.7 Mb), and explained 6.4, 4.5, 13.8 and 2.4% of the genetic variance for ∆BW, respectively. Twenty of the 40 coding genes that were annotated in these regions were potential candidate genes related to immune response, response to stress, and signalling pathways involved in cell/tissue growth (Table 1).

**Table 1:** Genomic regions associated with body weight deviation from expected growth (∆BW) after the vaccine challenge

SSC	Region^a^ (Mb)	SNP^b^	p-value	BF	%GV^c^	Candidate genes^d^
2	10.7–12.7	rs81240151	8.92E−07	695.9	6.4	*CD5*^e,g^, *CD6*^e,f,g^, *LPXN*^e,g^, *PRPF19*^f,g^, *TMEM109*^f,g^, *SLC15A*3^e,h^, *DTX4*^g^, *PTGDR2*^g^, *STX3*^g^, *MS4A2*^g^, *MS4A8*^g^, *MS4A10*^g^, *MS4A13*^g^, *MS4A15*^g^
8	12.8–14.8	rs322129753	3.35E−05	202.3	4.45	*LCORL*^h^, *SLIT2*^e,g^
9	135.8–138.7	rs81310044	3.22E−05	27.3	13.75	*IKZF1*^e^, *FIGNL1*^f,g^, *GRB10*^g,h^
		rs81323569	6.36E−06	134.6		
		rs81419253	3.26E−05	34.6		
		rs81419361	2.27E−06	395.9		
		rs81282478	1.61E−05	53.2		
		rs81334739	1.61E−05	52.7		
11	59.3–61.7	rs323869641	6.39E−05	34.4	2.35	*GPC5*^e^
		rs80828177	5.66E−05	84.3		

*SSC* *Sus scrofa* chromosome, *BF* Bayes factor

^a^Associated genomic region

^b^SNP identified as associated

^c^Proportion of the genetic variance of ∆BW explained by the associated genomic region

^d^Candidate genes involved in immune response^e^, response to stress^f^, signal transduction^g^, or growth^h^

### Increase in haptoglobin after the vaccine challenge

**Fig. 2:**
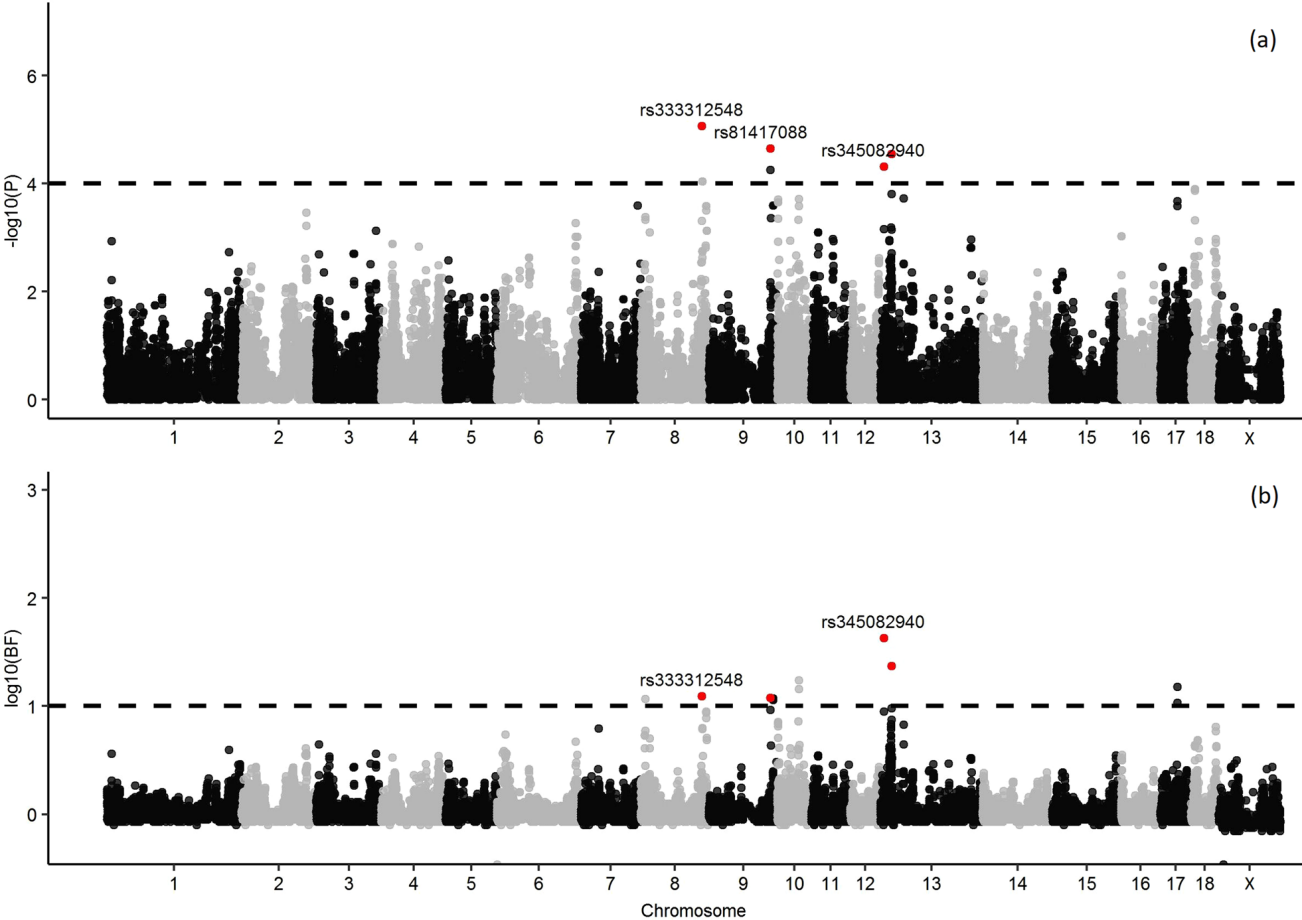
Manhattan plots for the genome-wide association analysis of the increase in haptoglobin four days following vaccination based on **a** single marker regression and **b** Bayesian multiple marker regression. The black dashed line represents the threshold of 0.0001 for p-values (**a**) and of 10 for the Bayes factor (**b**). Associated SNPs are highlighted in red

Seven SNPs, on SSC8, 9, and 13, were identified to be associated with ∆HP using SMR (Fig. 2a) and (see Additional file 2: Table S1) and 12 SNPs, on SSC8, 9, 10, 13, and 17, were identified using Bayes B (Fig. 2b) and (see Additional file 2: Table S2). Four of these SNPs were in common between the two approaches and therefore were considered as associated with ∆HP (Table 2). The genomic regions around these four SNPs explained 0.67% (SSC8, 123.8–125.8 Mb), 0.63% (SSC9, 123.9–125.9 Mb), 1.59% (SSC13, 5.7–7.7 Mb) and 2.14% (SSC13, 21.0–23.0 Mb) of the genetic variance for ∆HP. In total, 44 coding genes are annotated in the 1-Mb-windows around these four associated SNPs, and the 18 potential candidate genes that are related to immune or stress responses and signal transduction are in Table 2.

**Table 2:** Genomic regions associated with the increase in acute-phase protein haptoglobin (∆HP) four days after the vaccine challenge

SSC	Region^a^ (Mb)	SNP^b^	p-value	BF	%GV^c^	Candidate genes^d^
8	123.8–125.8	rs333312548	8.52E−06	12.4	0.67	*SMARCAD1*^e,f^, *ATOH1*^f^
9	123.9–125.9	rs81417088	2.23E−05	11.9	0.63	*RNASEL*^g^, *RGL1*^f^, *RGS16*^f^, *RGSL1*^f^, *RGS8*^f^, *LAMC1*^f^, *LAMC2*^f^, *NCF2*^g^
13	5.7–7.7	rs345082940	4.81E−05	42.4	1.59	*RAB5A*^g^, *KAT2B*^f^
	21.0–23.0	rs81326526	2.83E−05	23.6	2.14	*MLH1*^e,f,g^, *MYD88*^e,f,g^, *ARPP21*^e^*, PLCD1*^f^, *ACAA1*^f^, *LRRFIP2*^f^

*SSC* *Sus scrofa* chromosome, *BF* Bayes factor

^a^Associated genomic region

^b^SNPs identified as associated

^c^Proportion of the genetic variance of ∆HP explained by the associated genomic region

^d^Candidate genes involved in the response to stress^e^, or signal transduction^f^, or immune response^g^

### Body weight at 28 dpv

For BW_28_, Bayes B detected associations on 17 pig chromosomes, which highlights its polygenic nature (see Additional file 2: Table S2, Additional file 3: Figure S2). These SNPs were not detected using the SMR approach but three of them (rs81244626 on SSC1 at 250 Mb, rs81454578 on SSC15 at 118 Mb, and rs81326190 on SCC16 at 22 Mb) had P-values close to the threshold (see Additional file 3: Figure S2). The relevant genes on SSC15 (118 Mb) are *insulin-like growth factor-binding protein 2* (*IGFBP2*) and *5* (*IGFBP5*), which negatively regulate growth through the inhibition of insulin growth factor I [28, 29]. There was no overlap between the Bayes B regions for BW_28_ and the SNPs or regions identified for ∆BW.

## Discussion

The objective of this study was to identify genomic regions that are associated with resilience in pigs. Because resilience cannot be measured directly, over the last years, traits that reflect response to perturbations caused by stressors have been suggested as resilience indicators in several livestock species [3–7]. In previous work, we suggested ∆BW and ∆HP as novel resilience indicators in pigs, assuming that resilient pigs will quickly recover their growth performance and show high values of ∆BW and a minor activation of haptoglobin following a minor challenge with a commercial vaccine [8]. Some vaccines induce an episode of temporary growth arrest due to the anorexic effect of hyperthermia and inflammation [30], although the molecular mechanism of the growth depression is not fully understood. Vaccine challenges have been used to study variability in immune and production performance of livestock species and to predict their future response to infectious outbreaks [31–34]. In a period of immune stress, animals redirect nutrients that are destined for muscle synthesis and growth to the immune system to support increased functionality [35]. In this situation, haptoglobin production is initiated due to a rise in cytokine levels by monocytes and other tissue macrophages in response to injury [36]. The cytokine interleukin (IL) 6 that is produced in response to TNFα and IL-1β has been reported to be the major inducer for haptoglobin expression in liver and blood cells. In this study, the two resilience indicators, ∆BW and ∆HP, were measured in young pigs (12–16 weeks of age). This strategy for phenotype recording represents an advantage over phenotypes that are based on final production data, as data can be collected early in the life of the animal.

Association analyses between the phenotypes and genotypes were carried out using SMR and Bayes B approaches. Only SNPs that were identified to be associated with both methods were considered as associated with the phenotype. Correlations between estimates of associated SNP effects obtained from SMR and Bayes B were high and positive for both ∆BW (0.88) and ∆HP (0.84), which indicates that both approaches lead to similar results.

Four genomic regions were found to be associated with ∆BW and explained a relatively high proportion of that trait’s genetic variance. None of these regions overlapped with the genomic regions that were detected for BW_28_ by the Bayes B analysis (none were detected by the SMR analysis). This suggests that ∆BW not only reflects differences in BW_28_, but also differences in the animals’ ability to maintain a steady growth rate, in spite of the vaccine challenge. Immune responses have been described as the main pathways associated with changes in growth in chicken and pigs undergoing a lipopolysaccharide challenge [35, 37]. Similarly, in our experiment, the genomic regions associated with ∆BW included several genes that are directly involved in triggering an immune response (Table 1).

The genomic region on SSC2 (10.7–12.7 Mb) explained 6.4% of the genetic variance of ∆BW and harboured the most significant SNP (rs81240151), with a BF of 696. Several candidate genes map to this region, among which, *CD6 molecule* (*CD6*), *leupaxin* (*LPXN*), *prostaglandin D2 receptor 2* (*PTGDR2*) and *membrane spanning 4-domains A2* (*MS4A2*). The *CD6* gene encodes the T-cell differentiation antigen and regulates the adaptive immune system by promoting activation and proliferation of T cells [38, 39]. The *LPXN* gene negatively regulates the B-cell antigen receptor [40] and plays an important role in the B-cell immune response [41]. Furthermore, *PTGDR2* encodes the receptor for prostaglandin (PG) D_2_ and exerts pro- and anti-inflammatory properties [42]. The PGD_2_ protein is produced in the brain and regulates sleep and pain responses. Importantly, PGD_2_ is involved in sustaining the pyrogenic effect of PGE_2_ during inflammation [43]. In addition, a cluster of genes from the membrane-spanning 4A family (*MS4A2*, *MS4A8*, *MS4A10*, *MS4A13* and *MS4A15*) maps to this region. Many studies have reported the involvement of these genes in immune response [44, 45]. The most promising candidate gene is *MS4A2* (also known as *high-affinity immunoglobulin epsilon receptor subunit beta*, *FcERI*), which initiates the inflammatory response through the production of cytokines, particularly those leading to allergic reactions [46].

The SSC8 region that was found to be associated with ∆BW contains the *LCORL* locus, which has been associated with height in humans and with body size, growth rate and fat deposition in cattle, horses, and sheep (reviewed in [47, 48]). The molecular mechanism that underlies this association and the relationship between growth rate and resilience are yet unknown.

The genomic region on SSC9 (135.8–138.7 Mb) explained the largest proportion (13.75%) of the genetic variance for ∆BW. An important gene in this region is *IKAROS family zinc finger 1* (*IKZF1*), which encodes a transcription factor that has been implicated in B cell receptor signalling and differentiation of B and T cells [49, 50]. However, the *growth factor receptor bound protein 10* (*GRB10*) gene stands out as a strong candidate for this effect. This gene encodes a growth factor receptor-binding protein that interacts with insulin receptors and insulin-like growth-factor receptors that regulate responsiveness to insulin in a number of tissues, including in the thyroid gland and in myocytes [51]. The *GRB10* gene is ubiquitously expressed and exhibits a pattern of maternal or paternal imprinting, depending on the tissue and the species [52]. Moreover, sequence variants in the paternally-imprinted *GRB10* gene have been associated with birth weight in humans [53] and its expression pattern is known to respond to cytokines such as TNF [54].

The genomic regions on SSC8 (123.8–125.8 Mb), SSC9 (123.9–125.9 Mb), and SSC13 (5.7–7.7 and 21.0–23.0 Mb) that were found to be associated with ∆HP explained only a small portion of the genetic variance for this trait (from 0.63 to 2.14%). Combined together, these regions explained almost 5% of the genetic variance of ∆HP, while the identified associated regions for ∆BW explained more than 26% of its genetic variance. This could be expected since ∆HP is highly affected by environmental perturbations and has a relatively low heritability (0.16) [8]. Hepatic expression of acute-phase proteins is initiated by an increase in cytokines in response to infection, leading to a rise in the second wave of cytokines, which activates the release of the stored acute-phase proteins from blood monocytes and neutrophils. These acute-phase proteins act as immunomodulators that regulate the levels of cytokines, which, in turn, regulate the expression of acute-phase proteins. Haptoglobin also regulates the clearance of haemoglobin from the circulation by the macrophage-specific receptor CD163, thus preventing haemoglobin-induced oxidative damage [55]. The basic haptoglobin molecule is a tetrameric protein consisting of two α/β dimers. In pigs, the two subunits are encoded by a single gene located on SSC6 (15.0 Mb), which is not included in the relevant GWAS regions that were detected in our experiment and thus indicates that sequence variation at the pig haptoglobin gene has no major impact on its concentration, as previously described [56]. Relevant candidate genes in the genomic regions that were found to be associated with ∆HP are *ribonuclease L* (*RNASEL*) on SSC9 and *myeloid differentiation primary response gene 88* (*MYD88*) on SSC13*.* The *RNASEL* gene is an antiviral endoribonuclease that participates in innate immunity through regulation of the production of cytokines [57, 58]. Moreover, the genomic region on SSC9 (123.9–125.9 Mb) overlaps with a haptoglobin concentration quantitative trait locus detected at 0 and 10 dpv in 16 week-old PRRS virus vaccinated pigs [56]. The *MYD88* gene encodes an adaptor protein that is involved in the toll-like receptor/IL-1R receptor signalling pathway [59]. Activation of the latter induces the nuclear factor kappa (NF-kB) and mitogen-activated protein kinase (MAPK) signalling pathways, which are essential for the innate immune response [60]. In dendritic cells, MYD88 activates IL-6 [61], which is one of the main drivers of haptoglobin expression.

The current study identified several genomic regions that are associated with the resilience indicators ΔBW and ΔHP. The genomic regions associated with ∆BW and ∆HP do not overlap, which corroborates our previous findings that these indicators are not correlated and reflect different aspects of resilience [8]. Pigs were challenged with an attenuated Aujeszky vaccine to stimulate the immune response. Control and challenged pigs were reared in the same fattening batches under the same conditions. However, it is well documented that stress increases an animals’ susceptibility to disease. Hence, the resilience indicators do not only reflect specific response to the attenuated Aujeszky vaccine, but also capture a pig’s response to all the uncontrolled events that occurred during the experiment. Thus, the identified genomic regions are likely to be associated with a pig’s general resilience.

## Conclusions

Taken together, our results highlighted genomic regions that are associated with two resilience indicators (∆BW and ∆HP) in pigs that capture variation in growth depression and immune innate responses following vaccination. The associated regions harbour potential candidate genes that are related to immune response and signal transduction pathways that lead to growth. Our findings provide new insights into the genetic background of resilience. However, further analyses are necessary to validate the associations and confirm the role of the identified candidate genes.

## Supplementary Information

**Additional file 1: Figure S1.** Distribution of the deviation of body weight from the expected growth at 16 weeks of age (ΔBW) and the increase in haptoglobin four days after vaccination (ΔHP).

**Additional file 2: Table S1.** SNPs associated with the resilience indicators (ΔBW and ΔHP) using the single-marker regression approach. **Table S2** Title: SNPs associated with the resilience indicators (∆BW and ∆HP) and with BW_28_ using the Bayesian multiple marker approach. Description: ∆BW: deviation of body weight from the expected growth at 16 weeks of age after vaccination; ∆HP: increase in haptoglobin at four days after vaccination; BW_28_: observed body weight at 28 days after vaccination.

**Additional file 3: Figure S2.** Manhattan plots for the association analysis between observed body weight at 28 days after vaccination and the genotypes in pigs. Description: (**a**) single marker regression and (**b**) Bayesian multiple marker regression. The black dashed line represents the threshold of 0.0001 for p-values (**a**) and of 10 for the Bayes factor (**b**).

## Data Availability

All relevant data are included in the manuscript and its additional files. The datasets are available from the corresponding author on reasonable request.
