# Supplementary figures and images for: A genome-wide screen for resilient responses in growing pigs
Source: Genet Sel Evol. 2022 Jul 4;54:50. doi: 10.1186/s12711-022-00739-1 (PMC9251948; doi:10.1186/s12711-022-00739-1)

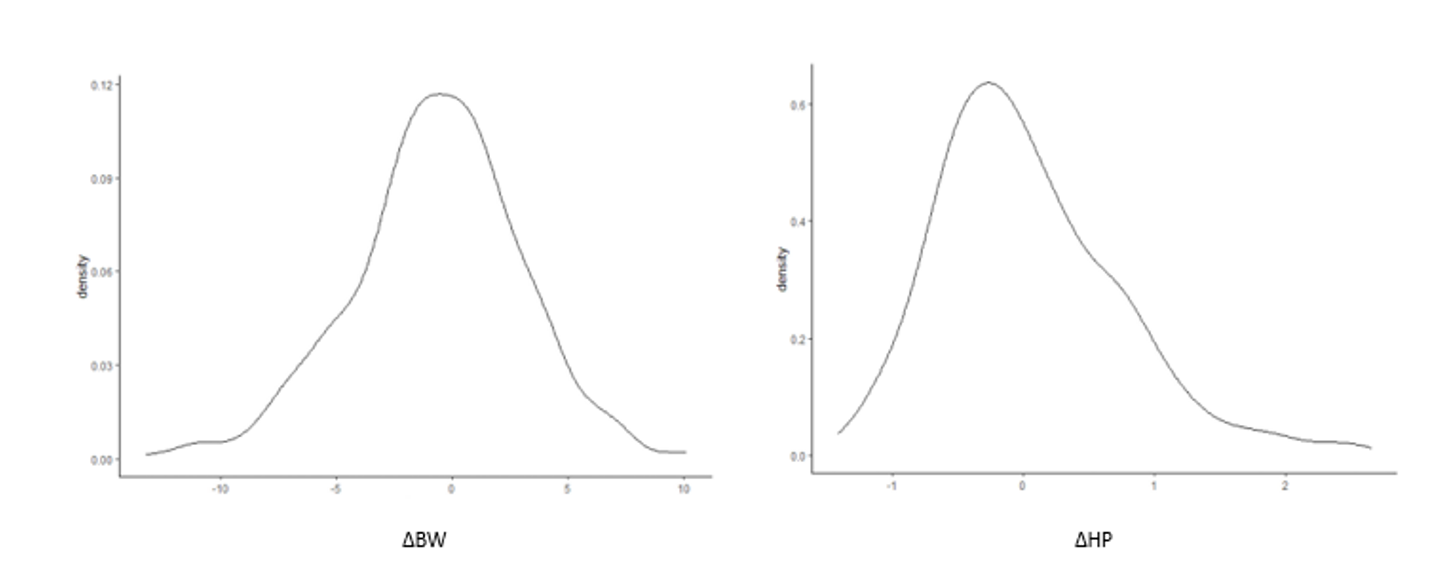

Supplement: Supplementary file 1 — Additional file 1: Figure S1. Distribution of the deviation of body weight from the expected growth at 16 weeks of age (ΔBW) and the increase in haptoglobin four days after vaccination (ΔHP). [file 12711_2022_739_MOESM1_ESM.png]

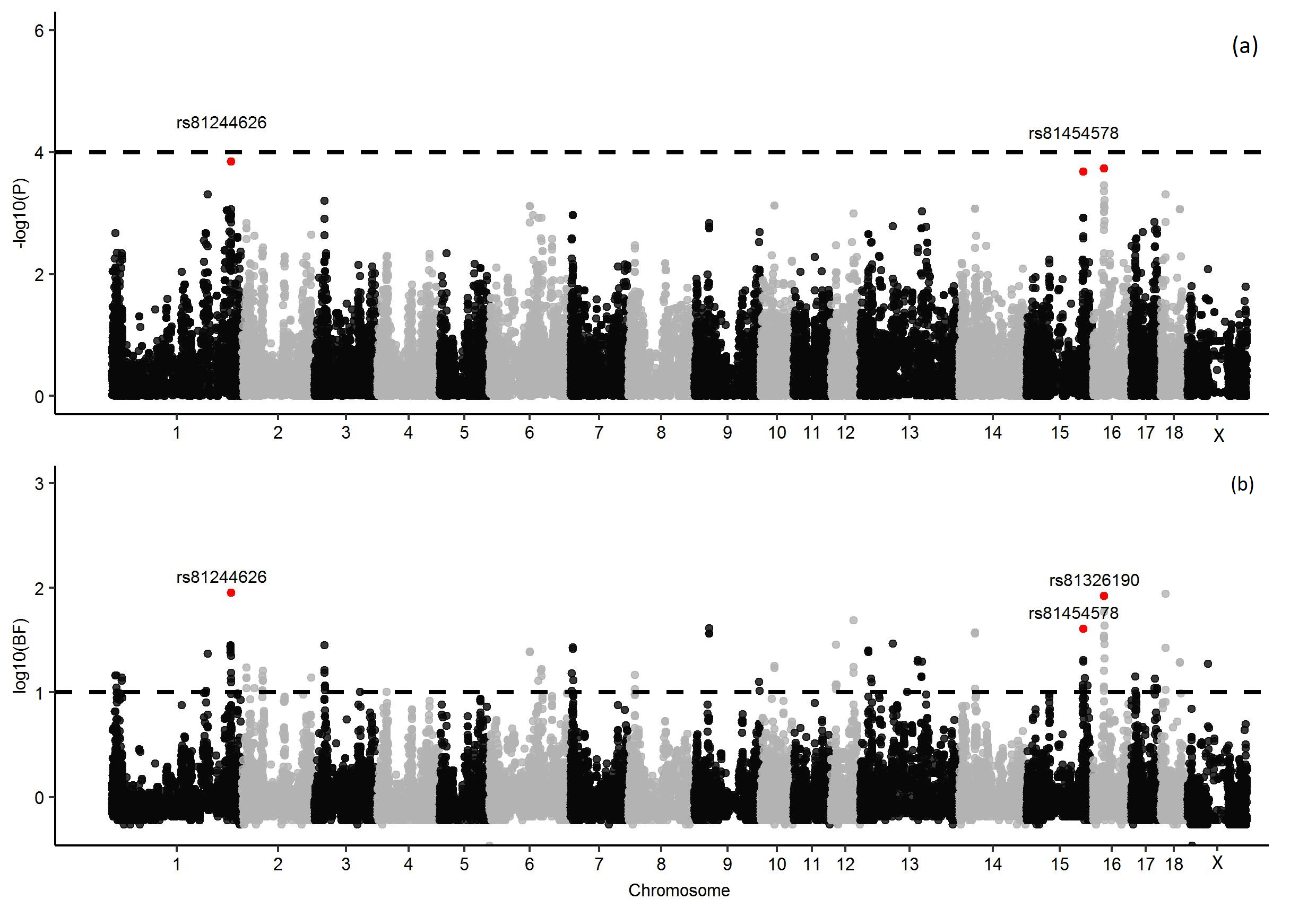

Supplement: Supplementary file 3 — Additional file 3: Figure S2. Manhattan plots for the association analysis between observed body weight at 28 days after vaccination and the genotypes in pigs. Description: (a) single marker regression and (b) Bayesian multiple marker regression. The black dashed line represents the threshold of 0.0001 for p-values (a) and of 10 for the Bayes factor (b). [file 12711_2022_739_MOESM3_ESM.png]
